# Growth Performance, Carcass Characteristics, and Meat Quality of Lambs Fed a High-Forage, Low-Starch, High-Oil Diet

**DOI:** 10.3390/foods15020193

**Published:** 2026-01-06

**Authors:** Eliana Jerónimo, Olinda Guerreiro, Andreia Silva, Patrícia Lage, Hélder Alves, João M. Almeida, Susana P. Alves, Rui J. B. Bessa, José Santos-Silva

**Affiliations:** 1 Centro de Biotecnologia Agrícola e Agro-Alimentar do Alentejo (CEBAL), Instituto Politécnico de Beja (IPBeja), 7801-908 Beja, Portugal; olinda.guerreiro@cebal.pt (O.G.); andreia.silva@cebal.pt (A.S.); patricia.lage@cebal.pt (P.L.); 2 MED—Mediterranean Institute for Agriculture, Environment and Development & CHANGE—Institute for Global Changes and Sustainability, Centro de Biotecnologia Agrícola e Agro-Alimentar do Alentejo (CEBAL), 7801-908 Beja, Portugal; 3 Centro de Investigação Interdisciplinar em Sanidade Animal (CIISA), Avenida da Universidade Técnica, 1300-477 Lisboa, Portugal; joaoalmeida@iniav.pt (J.M.A.); susanaalves@fmv.ulisboa.pt (S.P.A.); rjbbessa@fmv.ulisboa.pt (R.J.B.B.); jose.santossilva@iniav.pt (J.S.-S.); 4 Associate Laboratory for Animal and Veterinary Sciences (AL4AnimalS), Avenida da Universidade Técnica, 1300-477 Lisboa, Portugal; 5 Carlos e Helder Alves Sociedade Agro-Pecuária Lda., Funcheira, 7670-112 Garvão, Portugal; hmpalves@hotmail.com; 6 Instituto Nacional de Investigação Agrária e Veterinária, Polo de Inovação de Santarém, Quinta da Fonte Boa, 2005-048 Vale de Santarém, Portugal; 7 Faculdade de Medicina Veterinária, Universidade de Lisboa, Avenida da Universidade Técnica, 1300-477 Lisboa, Portugal

**Keywords:** animal growth, fatty acids, lamb, meat quality, oxidative stability

## Abstract

This experiment evaluated whether a high-forage, low-starch, and high-oil diet (experimental) could improve lamb meat fatty acid composition without compromising growth performance or overall meat quality, compared with a high-cereal diet typically used in intensive fattening systems (control). Ninety lambs were randomly assigned to six pens (fifteen animals/pen), with each diet provided to three pens for 32 days. Feed intake was monitored daily, and animal weight was monitored weekly. The feeding cost was also assessed. Four lambs per pen were slaughtered to assess carcass and meat traits. Average daily gain was unaffected by diet, but the experimental diet increased the feed conversion ratio. Kidney knob channel fat was higher in the experimental diet, while other carcass traits were unchanged. Meat sensory attributes and most physicochemical properties, including colour and lipid stability during storage, did not differ between diets. However, the experimental diet reduced meat pH and increased the proportions of *t*11–18:1, *c*9,*t*11–18:2, 18:2n-6, and 18:3n-3 in intramuscular fat, while *t*10–18:1 remained unchanged, and n-6 PUFA/n-3 PUFA ratio increased. A low-starch, high-forage, high-oil diet can be effectively used in lamb feedlots to enhance the intramuscular fat content of healthy fatty acids without compromising animal growth or meat characteristics, although it results in higher feeding costs.

## 1. Introduction

The intensive lamb fattening system, based on confinement and high-energetic concentrate diets, has been increasingly adopted in Portugal to the detriment of the traditional pastured-based extensive production system. In several regions of the world, including the Mediterranean, climate change has made it increasingly difficult to fatten lambs exclusively on pasture and forages. In addition to the difficulty in the availability of pasture and forage throughout the year, factors such as market demand have promoted the transition to intensive production systems that ensure rapid growth, high productivity, and standardization of the final product [1], which is appreciated by the lamb meat industry.

However, this change in the lamb meat production system raises a challenge for the product’s nutritional quality, with particular concern regarding its fatty acid (FA) composition. High-cereal diets, commonly used in the intensive lamb finishing systems, are associated with an alteration in the ruminal biohydrogenation (BH) pathway (known as the *t*10-shift), which favours the ruminal production of *t*10–18:1 instead of vaccenic acid (*t*11–18:1) [2,3]. This shift results in a reduction in the rumen outflow of *t*11–18:1, and consequently, its availability for endogenous conversion into rumenic acid (*c*9,*t*11–18:2) [2]. As a result, lambs fed cereal-rich concentrate diets have higher intramuscular fat levels of *t*10–18:1, whereas animals reared under pasture-based or forage-rich feeding systems show greater levels of *t*11–18:1 and *c*9,*t*11–18:2 [4,5]. The specific effects of *trans* FA on human health are not yet fully established. However, current evidence suggests that *t*10–18:1 exerts detrimental effects, whereas *t*11–18:1 and *c*9,*t*11–18:2 have been linked to beneficial health properties [4,6,7,8].

Nutritional strategies aimed at limiting the *t*10-shift and enhancing the *c*9,*t*11–18:2 content in ruminant fat, while sustaining high levels of animal productivity, have been the focus of extensive research in recent decades. Results show that diets containing 40% high-quality forage, where low-starch agro-industrial by-products replace part of the cereals and are supplemented with PUFA-rich vegetable lipid sources, are an effective strategy to limit the occurrence of the *t*10-shift and enhance the levels of *t*11–18:1 and *c*9,*t*11–18:2 in ruminant fat [9]. At the same time, this feeding strategy allows the maintenance of high animal performance. Therefore, we hypothesize that a diet with these characteristics can be applied in lamb feedlots to improve the FA composition while maintaining animal performance indicators, carcass characteristics, and meat quality parameters. However, the effectiveness of this feeding strategy has not been compared with the conventional diet applied in an intensive lamb finishing system, which is essential to validate its applicability under commercial production conditions.

So, the objective of this study was to evaluate, in a lamb feedlot, the effect of a diet composed of a 60:40 concentrate-to-forage ratio, supplemented with 6% of soybean oil and with partial replacement of cereals with dehydrated pulps of citrus and sugar beet and soybean hulls, compared with a conventional high-cereal concentrate diet, on the growth performance, carcass traits, and physicochemical and sensory meat quality.

## 2. Materials and Methods

### 2.1. Animals, Diets, and Experimental Design

The present experiment was conducted on a lamb fattening farm located in Ourique, in the Baixo Alentejo region of Portugal, in accordance with the procedures approved by the Animal Care Commission of the Instituto Nacional de Investigação Agrária e Veterinária I. P. (INIAV I.P.; REF: ORBEA-INIAV-2020/01) and in compliance with the European Union Directive 2010/63/UE [10].

Ninety Merino Branco ram lambs, approximately 60 days of age and 21.2 ± 2.42 kg, were randomly divided into 6 indoor pens (15 animals per pen) with straw bedding. Two dietary treatments were applied in this experiment: (1) a conventional diet based on commercial concentrate (control, Ovicor Medio G, Nanta Portugal S.A., Alverca do Ribatejo, Portugal); (2) an experimental diet consisting of a high-fibre, low-starch, and high-lipid diet (experimental). Both diets included wheat straw, which was provided as 10% of the total diet. The label on the commercial concentrate indicates that it contains barley, corn, wheat, soluble dry corn distillates, soybean hulls, extracted dehydrated soybean meal, corn kernels, beet molasses, and soybean oil. The experimental diet consisted of 40% dehydrated Lucerne, 17.5% low-starch agro-industrial by-products (dehydrated pulps of citrus and sugar beet, and soybean hulls), 17.6% cereals (maize and wheat), 14% protein sources (soybean and sunflower meals), and 6% soybean oil. Table 1 presents the ingredients and the proportions used in the formulation of the experimental diet. The experimental diet was produced in the Feed Compound Unit of Polo de Inovação da Fonte Boa—INIAV I. P.

Samples of both the control and experimental feeds were collected weekly and pooled into 3 samples, which were used for chemical characterization (Table 2). Both diets were offered ad libitum, once daily at approximately 9:00 am, considering 10% refusals in each pen. Animals underwent a 7-day adaptation period to the facilities and diets, followed by a 32-day experimental phase. The experiment duration was set according to commercial practices of intensive lamb fattening in Portugal, where short fattening periods are used. During the experiment, feed intake was monitored daily, and the animals’ weights were recorded weekly before feed distribution.

To estimate the feeding cost per kg live weight gain, the feed conversion ratio was multiplied by the unit cost of the respective diet. Diet unit costs were calculated using market prices in Portugal during June–July 2022, corresponding to 478.00 €/t for the control diet and 442.62 €/t for the experimental diet.

### 2.2. Slaughter, Carcass Evaluation and Sampling

At the end of the experiment, 4 lambs from each pen (12 lambs per dietary treatment) were randomly selected and transported to the facilities of INIAV I. P.—Polo de Inovação da Fonte Boa, Vale de Santarém, Portugal, located 220 km from the lamb fattening farm. The lambs were kept in this facility for approximately 20 h with access to control or experimental diets and clean water. Immediately before transport to the experimental slaughterhouse at INIAV I.P.—Polo de Inovação da Fonte Boa, located approximately 400 m away, the lambs were weighed. Lambs were slaughtered by exsanguination after electrical stunning, and within a maximum period of 1 h after arrival at the slaughterhouse. For rumen pH analysis, the rumen contents of each lamb were collected immediately after slaughter, homogenized, and strained through 4 layers of cheesecloth. The pH of rumen fluid (ca. 50 mL) was measured using a pH metre (Metrohm 744, Herisau, Switzerland).

After skinning, the hot carcass weight was recorded. After storage at 2 °C for 24 h, cold carcass weight was assessed, and carcasses were evaluated for conformation (six classes—S, E, U, R, O, P) and fat cover (five classes—1, 2, 3, 4, 5) using the European Union classification system for lamb carcasses weighing more than 13 kg [12]. Subsequently, carcasses were stored at 2 °C for an additional period of 24 h.

Carcasses were prepared, and muscle samples were collected 48 h after slaughter. The carcass preparation began with the removal of the kidney knob channel fat (KKCF) and kidneys, which were then weighed, followed by dividing the carcass into two parts along the vertebral column. The shoulder, from the left side of the carcass, was collected for dissection into muscle, bone, and subcutaneous and intermuscular fat. The vacuum-packed shoulders remained at −20 °C for 2 months until dissection. The loins, containing the *Longissimus lumborum* (LL) muscle, on both sides of the carcass, were vacuum-packed and stored for 7 days at 2 °C. After this short-chilled period of 7 days at 2 °C, the meat samples were then frozen at −20 °C, simulating real storage conditions, until used for shear force, cooking losses, and sensorial analysis. The left loins were used for shear force and cooking loss analysis, while the right loins were used for sensory analysis.

For the analysis of muscle pH and chemical composition, including FA composition, a portion of the *Longissimus thoracis* (LT) muscle was minced using a food processor (Moulinex-123 A320R1, Group SEB Portugal Lda, Lisbon, Portugal), after removal of the epimysium, and then vacuum-packed and stored at −80 °C. From another portion of the LT muscle, three slices approximately 1.5 cm thick were prepared to assess lipid oxidation and colour stability during storage for 0, 4, and 7 days. The meat slice corresponding to day 0 of storage was vacuum-packed and frozen (–80 °C) immediately after colour determination. The remaining 2 slices were individually placed on polystyrene trays, which were overwrapped with an oxygen-permeable film, and stored for 4 and 7 days, respectively. During this time, samples were kept in a refrigerated chamber at 2 °C with light intensity ranging from 265 to 270 Lux. Colour coordinates were measured after 1 h of blooming in day 0 samples, and 1 h after film removal in samples stored for 4 and 7 days. Following colour measurement, all samples were vacuum-packed and frozen at −80 °C.

All meat samples were vacuum-packaged in polyamide and polyethylene (20/70) food bags (Termofilm embalagens técnicas Lda., Vila Nova de Famalicão, Portugal), using a vacuum machine (AUDIONVAC VMS 123 G, Audion Packaging Machines, Weesp, The Netherlands).

### 2.3. Analytical Determination

#### 2.3.1. Feed Chemical Composition

The control and experimental diets were characterized in terms of dry matter (DM) [13], ash [14], ether extract [15], crude protein [16], sugar, starch [17], neutral detergent fibre (NDF), acid detergent fibre (ADF), and acid detergent lignin (ADL) [18] contents. Total phenol content and antioxidant capacity were evaluated in the extract prepared from both diets using an acetone:water (70:30, *v*/*v*) solution, following the procedure described by Makkar [19]. Total phenol content was quantified using the Folin–Ciocalteu method [19], with tannic acid as the standard. Antioxidant capacity was assessed using two assays—ferric reducing antioxidant power (FRAP) and trolox equivalent antioxidant capacity (TEAC), conducted as described by Luciano et al. [20]. For FA composition analysis, feed lipids were directly transesterified according to Sukhija and Palmquist [21]. The resulting FA methyl esters (FAME) were analyzed using a Shimadzu GC 2010-Plus (Shimadzu, Kyoto, Japan) with flame ionization detection (FID) and a SP-2560 capillary column (100 m × 0.25 mm, 0.20 µm film thickness, Supelco, Bellefonte, PA, USA). The chromatographic conditions are described in Vítor et al. [22].

#### 2.3.2. Chemical Composition, Physical and Sensory Properties of Meat

The LT muscle moisture and crude protein contents, and pH were determined according to the international standard methods ISO 1442 [23], ISO 2917 [24], and ISO 2917 [24], respectively. For the analysis of intramuscular fat content and composition, muscle samples were previously freeze-dried for 48 h at −55 °C using a ScanVac Coolsafe freeze dryer (LaboGene, Denmark). Lipids were then extracted following a modified version of the Folch et al. [25] method, in which chloroform and methanol (2:1, *v*/*v*) were replaced with dichloromethane and methanol (2:1, *v*/*v*). Lipid extracts were transesterified into FAME using a combined basic and acid catalysis as described by Cruz-Hernandez et al. [26], and subsequently analyzed by gas chromatography using a Shimadzu GC 2010-Plus (Shimadzu, Kyoto, Japan) with flame ionization detection (FID) and SP-2560 capillary column (100 m × 0.25 mm, 0.20 µm film thickness, Supelco, Bellefonte, PA, USA). Nonadecanoic acid (19:0, 1 mg/mL) was used as an internal standard. The chromatographic conditions are described in Vítor et al. [22].

For the evaluation of meat shear force and cooking loss, the loins were previously thawed for 24 h at 2 ± 1 °C. After thawing, bone and fat residues were removed, and the samples were weighed before being cooked in an electric oven at 170 ± 5 °C. The internal temperature was monitored using a T-type thermocouple (Thermometer, Omega RDXL4SD, Manchester, NH, USA), and samples were withdrawn from the oven once a core temperature of 71 °C was reached. Subsequently, they were rinsed with cold water to remove precipitated exudates and to initiate rapid cooling, stored at 4 ± 0.5 °C for 20 h, and then reweighed. Cooking loss was calculated as the difference between the weight of the meat samples before and after cooking, and the results were expressed as a percentage of the initial weight. The shear force was determined in LL muscle subsamples with an area of 1 cm^2^ and 3 cm of length, using a Texture Analyzer (TA-XT2 Texture Analyzer; Stable Micro Systems, Surrey, UK) with a 30 kg compression load cell. Shear force measurements were obtained by cutting the samples across the muscle fibres at a crosshead speed of 2 mm/s, along 25 mm. Twenty measurements were performed for each sample.

The sensory evaluation of lamb meat was carried out over four sessions by nine trained members according to the criteria established in ISO 8586-1:1993 [27]. The sensorial panel consisted of four men and five women, aged between 25 and 65 years, who agreed to participate in sensorial analysis and provided informed consent. In each session, samples from the two dietary treatments were randomly selected for evaluation. For sensorial analyses, LL muscle samples were prepared and cooked according to the procedure previously used for shear force and cooking loss determinations. After cooking, samples were stabilized for 10 min at 40 °C and subsequently cut into cubes (1 × 1 × 1 cm). In a pre-heated disposable Petri dish, two cubes of meat were placed, then covered and maintained at 40 °C until evaluation, which was conducted within 30 min of sample preparation. The lamb meat was assessed for juiciness, tenderness, odour intensity, flavour intensity, flavour acceptability, and overall acceptability using a structured numerical scale from 1 to 6, where 1 indicated ‘extremely dry, tough, lacking odour, lacking flavour, and unpleasant’, and 6 indicated ‘extremely juicy, tender, odorous, flavoursome, and pleasant’.

#### 2.3.3. Colour and Lipid Stability of Meat

Colour coordinates were determined in meat samples refrigerated at 2 °C over 0, 4, and 7 days with a Minolta CR-400 chromometer (Konica Minolta, Tokyo, Japan) according to the CIE system L*, a*, b*, in which L* is lightness, a* is redness, and b* is yellowness. Measurements were performed using the C illuminant, standard 2° observer, and a 10 mm aperture. The colorimeter was calibrated each day, before measuring the colour coordinates, using a Minolta white standard calibration plate (Y = 87.7, x = 0.3154, y = 0.3227). Three measurements were recorded for each sample. Using colour coordinates, Hue angle (H*) (1) and colour saturation (chroma, C*) (2) were calculated.

H* = tan^−1^ (b*/a*) × (180/π) (1)

C* = (a*^2^ + b*^2^)^1/2^ (2)

Meat colour variation between days 4 and 0 and between days 7 and 0 of storage was assessed by calculating the colour stability index (ΔE, 3).

ΔE = (L*_(4 or 7)_ − L*_0_)^2^ + (a*_(4 or 7)_ − a*_0_)^2^ + (b*_(4 or 7)_ − b*_0_)^2^)^1/2^ (3)

Meat lipid oxidation during storage was evaluated by quantifying 2-thiobarbituric acid reactive substances (TBARS), following the methodology described by Grau et al. [28].

### 2.4. Statistical Analysis

This trial followed a completely randomized design, in which the pen was considered the experimental unit (*n* = 3). Data were analyzed using the MIXED procedure of SAS 9.4 (SAS Institute Inc., Cary, NC, USA), considering the diet as a fixed effect, the pen as a random effect, and the animal within each pen as subsampling. The variance heterogeneity was tested, and if significant (*p* < 0.01), the group option of the repeated statement was included in the model. The level of statistical significance was set at *p* < 0.05.

The average daily gain (ADG) of lambs was estimated using the random intercept regression model, considering the live weight recorded over the experiment. For the analysis of daily intake of DM and nutrients, a model that also included the day of experiment as a repeated measure was used, considering a first-order autoregressive (AR(1)) covariance structure (selection based on Akaike information criteria (AICC)). The model used to analyze live slaughter weight and hot and cold carcass weights included initial live weight as a covariate. Additionally, hot carcass weight was included as a covariate in the models used to analyze the percentage of kidney knob channel fat in the carcass, as well as the tissue composition of the shoulder. Data from carcass classification were analyzed using the GLIMMIX procedure in SAS using a binary distribution. Tasters and seasons were included as random effects in the model used to analyze meat sensory attributes.

Data from meat colour and lipid oxidation were analyzed using a model that included the diet and storage time (0, 4, and 7 days) as fixed effects and the diet × storage time interaction. This model accounted for repeated measures over time for each sample, utilizing a first-order autoregressive (AR(1)) covariance structure. However, the diet × storage time interaction was removed from the model since it was not significant for any of the variables analyzed.

## 3. Results

### 3.1. Growth Performance and Feed Intake

Dietary treatments did not affect slaughter live weight and average daily weight gain, averaging 31.4 kg and 333 g/day, respectively (Table 3). However, compared to the control diet, the experimental diet increased the feed conversion ratio (*p* = 0.002, +31%) and the feeding cost per kg of weight gain (*p* = 0.006, +21%).

The experimental diet increased the (*p* < 0.05) daily intake of DM (+24%), crude protein (+5%), ether extract (+405%), NDF (+137%), ADF (+354%), ADL (+468%), ash (+90%), and total phenols (+220%) compared to the control diet (Table 3). Conversely, the experimental diet reduced the daily intake of starch by 57% compared to the control diet, while sugar and metabolizable energy intake did not differ between the control and experimental diets. The daily intake of FA (16:0, 18:0, *c*9–18:1, *c*11–18:1, 18:2n-6, and 18:3n-3) was also higher (*p* < 0.001) in the experimental diet than in the control one.

The post-mortem rumen pH (*p* < 0.001) was higher in the experimental diet than in the control diet.

### 3.2. Carcass and Meat Quality Traits

**Figure 1:**
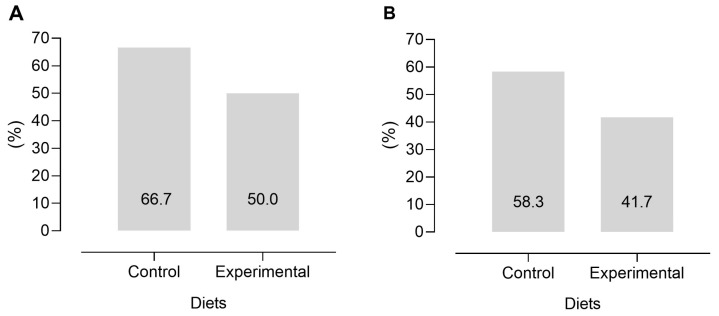
Probability of the occurrence of carcasses graded as R (good) for conformation (*p* = 0.419, SEM = 0.144) (**A**) and as 3 (average) for the fat cover score (*p* = 0.415, SEM = 0.132) (**B**) in lambs fed a conventional concentrate diet (control) or a high-fibre, low-starch and high-lipid diet (experimental). Data from 12 lambs per diet (4 lambs per pen and 3 pens per diet). Least squares means are presented in each bar.

Dietary treatments did not influence the hot and cold carcass weight and dressing percentage, which averaged 15.5 kg, 15.0 kg, and 48.5%, respectively (Table 4). The tissue composition of the shoulder also remained unaffected by diets, averaging 588, 222, 107, and 78 g/kg for muscle, bone, intermuscular fat, and subcutaneous fat, respectively. On the other hand, the experimental diet increased the kidney knob channel fat percentage than the control diet. All carcasses from both diets were graded as O (fair) or R (good) for conformation and as 2 (slight) and 3 (average) for the fat cover, and the dietary treatments did not affect carcass conformation (*p* = 0.419) and fat cover (*p* = 0.425) (Figure 1).

Except for the pH of meat, which was higher in the control diet than in the experimental diet (*p* = 0.032), the other meat quality parameters were not differ between the two diets, averaging 244 g/kg for DM, 206 g/kg for protein, 12.3 g/kg for intramuscular fat, 30.5 g/100 g for cooking losses and 37.1 N for shear force. Dietary treatments also did not affect meat sensory attributes.

### 3.3. Meat Colour and Lipid Stability

Meat colour parameters did not differ between control and experimental diets (*p* > 0.05, Table 5). Regardless of the diet, the values of b*, C*, and H* increased over the storage time, while a* decreased (*p* < 0.001). The value of L* remained unchanged throughout the storage period. Meat lipid oxidation did not differ between diets (*p* = 0.606) but increased over storage time (*p* < 0.001).

### 3.4. Fatty Acid Composition of Intramuscular Fat

The sum of linear chain saturated fatty acids (LC-SFA) was 6.62% lower in the experimental diet than in the control diet (*p* = 0.034, Table 6). This reduction in the experimental diet was due to a decrease in odd-chain fatty acids 15:0 (*p* = 0.007) and 17:0 (*p* = 0.001), while the levels of other LC-SFA did not differ between diets (Table 5). Among the branched-chain fatty acids (BCFA), only iso-15:0 (*p* = 0.006), iso-18:0 (*p* < 0.001), and anteiso-17:0 (*p* = 0.031) were affected by diets, with a lower proportion of iso-15:0 and higher proportions of iso-18:0 and anteiso-17:0 in the control diet than in the experimental diet. The experimental diet reduced the sum of *cis*-monoenoic FA by 21.6% compared to the control diet (*p* < 0.001), due to reductions in most individual *cis*-monoenoic FA, except *c*9–14:1, which remained unaffected. Conversely, the sum of n-6 PUFA was higher in the experimental diet than in the control one (*p* = 0.031), particularly due to the increase in the proportion of the 18:2n-6 (*p* = 0.007, +84%). The 20:2n-6 (*p* = 0.023) was also higher in the experimental diet, while the 18:3n-6 (*p* = 0.032) was higher in control-fed animals. The levels of other individual n-6 long-chain PUFA (n-6 LC-PUFA) and the sum of n-6 LC-PUFA did not differ between diets. Regarding n-3 PUFA, the experimental diet increased the proportion of 18:3n-3 (*p* < 0.001, +77%), but decreased the proportions of 20:5n-3 (*p* = 0.002) and 22:6n-3 (*p* = 0.002), leading to a lower sum of n-3 long-chain PUFA (n-3 LC-PUFA) in the intramuscular fat of lambs fed the experimental diet (*p* = 0.034, −32%). However, the sum of all PUFA was 58% higher in the experimental diet than in the control diet (*p* = 0.016).

**Figure 2:**
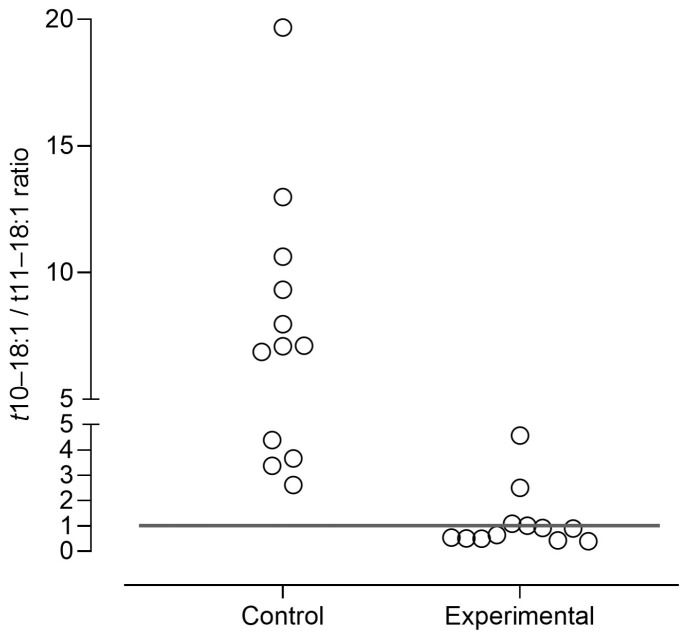
Individual results for *t*10–18:1/*t*11–18:1 ratio in intramuscular fat from lambs fed a conventional concentrate diet (control) or a high-fibre, low-starch and high-lipid diet (experimental). Data from 12 lambs per diet (4 lambs per pen and 3 pens per diet).

Regarding the ruminal biohydrogenation intermediates (BHI, Table 7), the experimental diet increased the proportions of all BHI, except *t*10–18:1, *t*13–18:1, and *t*8,*c*13-/*c*9,*t*15–18:2, which remained unchanged. The experimental diet increased the sums of 18:1 BHI and 18:2 BHI by 119% and 292%, respectively. In lambs fed the control diet, *t*10–18:1 was the main *trans*-FA, while in lambs fed the experimental diet, both *t*10–18:1 and t11–18:1 were abundant, with *t*11–18:1 slightly higher than *t*10–18:1 (31.6 vs. 28.4% of total FA, respectively). It should be noted that *c*9,*t*11–18:2 was the main dienoic BHI in both dietary treatments, with its proportion being 398% higher in the experimental diet compared to the control diet. All lambs fed the control diet had a *t*10–18:1/*t*11–18:1 ratio above 1, the threshold that defines the occurrence of *t*10-shift (Figure 2). In contrast, among lambs on the experimental diet, only 4 animals had a *t*10–18:1/*t*11–18:1 ratio above 1, and for 2 of them, the ratio was only slightly above 1.

Table 8 presents the concentrations of various individual FA and FA sums and indices, expressed in mg/100 g of meat, which are relevant from a nutritional perspective. The experimental diet increased the intramuscular fat contents of *t*11–18:1 (*p* = 0.008) and *c*9,*t*11–18:2 (*p* = 0.007) compared to the control diet. Additionally, the intramuscular fat contents of 18:2n-6 and 18:3n-3 were higher in the experimental diet (*p* < 0.001) than in the control diet, while the contents of 20:5n-3 (*p* = 0.016) and 22:6n-3 (*p* < 0.001) decreased.

The experimental diet also increased the total content of PUFA (*p* < 0.001), n-6 PUFA/n-3 PUFA ratio (*p* = 0.020), and health-promoting index (*p* = 0.035), and reduced the atherogenicity index (*p* = 0.037). The sums of LC-SFA, BCFA, hypocholesterolemic/hypercholesterolemic (HH) ratio, and thrombogenicity index were not affected by the diet (*p* > 0.05).

## 4. Discussion

In Portugal, as in other Mediterranean countries, lamb finishing is often performed under intensive systems in which animals are confined and fed cereal-based diets. Although such high-energy diets promote growth, feed efficiency, and overall carcass and meat quality, they may compromise the nutritional value of meat by increasing the FA associated with detrimental health effects and reducing those with potential health benefits [2]. In this context, there is a need to offer viable alternative feeding strategies for fattening lambs in intensive systems that sustain high animal growth rates and desirable carcass traits, while improving the meat’s nutritional profile. Thus, the present study evaluates a diet that has been appointed as a good strategy for achieving this goal, containing 40% high-quality forage, with partial replacement of cereals by low-starch agro-industrial by-products and supplemented with vegetable lipid sources rich in PUFA. This alternative dietary approach was compared to the conventional diet to assess its effectiveness and practical applicability under commercial conditions, highlighting its potential advantages and limitations.

### 4.1. Growth Performance and Feed Intake

The feeding strategy explored in the present work resulted in a diet with a chemical composition that differs significantly from the conventional diet used in intensive lamb fattening systems. This difference may raise concerns regarding the potential impact of the experimental diet on animal performance, as well as on carcass and meat quality. The experimental diet contained higher levels of fibre (+ 91% of NDF) and ether extract (+307%), and lower levels of starch (−66%), crude protein (−15%), and metabolizable energy (−21.6%), which can affect the voluntary feed intake, as well as the ruminal fermentation and the feed digestibility. Changes in the ruminal microbial ecosystem and fermentation patterns are expected due to the higher levels of NDF in the experimental diet, promoting the cellulolytic bacteria and acetate production [32]. On the other hand, fatty acids, particularly unsaturated FA, which have antimicrobial effects in the rumen, can cause adverse effects on ruminal fermentation and feed digestibility [33]. In fact, negative interactions between dietary oil and rumen digestibility of nutrients have been reported [34]. Depression of the feed intake was also reported in lambs fed lipid-supplemented diets [29,35], although this effect is not consistent [36,37]. However, despite the distinct chemical composition of the two diets and the expected changes in rumen metabolism and ecosystem, both diets led to similar ADG, slaughter live weight, and carcass weight. Furthermore, ADG was high in both diets and within the expected growth rate for Merino lambs in a similar weight range and fed high-energy diets [38]. However, the high growth performance observed in lambs fed the experimental diet was accompanied by an increase in DMI (+265 g/d) and, consequently, a higher feed conversion ratio. The higher DMI observed in lambs fed this diet may be the result of adaptation to the different energy densities of the diets. The voluntary feed intake is strongly influenced by the diet energy density [39]. Thus, the higher dry matter intake (DMI) observed on lambs fed the experimental diet may be due to an adaptive response mechanism to the lower metabolizable energy in this diet, resulting in similar metabolizable energy intake between the control and experimental diets. This adjustment in energy intake, enabled by the higher DMI in the experimental diet, likely allowed the lambs receiving this diet to achieve similar growth to those fed the control diet. Therefore, our results showed that higher fibre and fat levels in the experimental diet did not limit feed intake. This result is consistent with increased feed intake in lambs fed an oil-supplemented diet when dehydrated Lucerne is provided in ground form, in contrast to the reduction in intake observed when Lucerne was supplied in larger particles (chopped) [40].

Feeding costs represent a substantial part of global livestock production expenses, and any variation in production costs arising from feed cannot be neglected. The unit cost of the experimental diet was 7.4% lower than that of the control diet. However, the higher feed conversion ratio in the experimental diet led to an increased feed cost per kg of weight gain, which may limit its practical application in commercial production systems. In lamb-fattening systems, feeding cost is a major determinant of profitability, so an increase in cost per unit of weight gain directly compromises the viability of adopting this feeding strategy on a large scale.

### 4.2. Carcass Traits

Carcass traits were not influenced by dietary treatments, except for the percentage of kidney knob channel fat (KKCF), which was higher in the experimental diet. This result is consistent with other studies that reported an increase in KKCF deposition in the lamb carcasses when diets are supplemented with oil [29,36]. However, dietary oil supplementation did not affect shoulder tissue composition or intramuscular fat content. Additionally, carcass fat cover scores were similar between diets, with most carcasses classified as having average fat cover. In both diets, the meat’s intramuscular fat content (12.3 g/kg in fresh meat) was lower than values reported for Merino lambs of similar slaughter age and carcass weights [40]. Intramuscular fat deposition is affected by multiple factors [41]. Differences in diets or factors specific to each group of animals (e.g., origin and age) may help to explain the discrepancy in results.

### 4.3. Meat Quality

Regarding meat quality parameters, only pH was affected by the dietary treatments, with lower values observed in meat from lambs fed the experimental diet. According to the present work, Francisco et al. [40] also reported a linear decrease in meat pH when barley was progressively replaced in the lambs’ diet with a mixture of the same agro-industrial by-products used in the present work. Despite the reduction, the meat pH values in both dietary groups remained below 5.8 and, therefore, within the range considered normal for lamb meat [42]. Furthermore, the meat produced under both dietary treatments can be considered tender, as shear force values were below the threshold commonly used to define tender lamb meat (49 N) [43]. The processing conditions applied—chilling for 7 days at 2 °C followed by freezing at −20 °C, may have contributed to the low shear force values observed in the meat from both dietary treatments [44,45].

The most crucial factor influencing consumer acceptance and purchasing decisions of meat is colour. Regardless of dietary treatment, meat colour parameters varied during refrigerated storage, reflecting meat browning as indicated by increased b* and H* and decreased a* [46]. However, despite these changes over the storage period, both L* and a* values remained above and close, respectively, to the threshold value considered acceptable by consumers (>44 for L* and >14.5 for a*) [47]. Additionally, the ∆E, which provides a global assessment of meat colour variation over 7 days of storage, did not differ between diets. Furthermore, the colour changes appear not to be detectable by the consumers, once the ∆E values are below 6 [48].

The FA composition of intramuscular fat influences several aspects of the meat quality, contributing significantly to its oxidative stability. The higher PUFA content in meat from lambs fed the experimental diet may create favourable conditions for lipid oxidation, since PUFAs are more prone to oxidation [49]. Despite the higher PUFA contents in the meat from the experimental diet, lipid oxidation levels did not differ between diets. The experimental diet had higher total phenol content and antioxidant capacity, evaluated by FRAP and TEAC assays, than the control diet, which could have contributed to neutralizing the higher oxidative pressure created by the higher PUFA content in meat from the experimental diet. The protective effect against meat lipid oxidation when antioxidant-rich by-products are included in lamb diets has been widely reported [50,51,52]. Regardless of diet, lipid oxidation levels in the meat increased over the storage time. After 7 days of refrigerated storage, TBARS values slightly exceeded the acceptable threshold of 1 mg MDA/kg meat reported for lamb meat by Ripoll et al. [53]. However, values remained below the threshold value of 2 mg MDA/kg meat for sensory perception of lipid oxidation in beef [54].

Health concerns associated with ruminant fat intake are primarily due to its high saturated fatty acids (SFA) content, but also to the presence of *trans*-FA, as the specific *trans*-FA isomers present in ruminant fat have been linked to adverse effects on human health. In this context, lamb meat from intensive production systems has received particular attention, as it often contains higher levels of *t*10–18:1 and lower concentrations of *t*11–18:1 and *c*9,*t*11–18:2, due to the occurrence of the *t*10-shift. Therefore, the present study was specifically designed to assess whether a high-fibre, low-starch, and high-lipid diet is effective in the prevention of *t*10-shift occurrence and improve the FA profile in lamb meat, while maintaining animal performance indicators, carcass characteristics, and overall meat quality, compared to a conventional diet used in intensive fattening systems. Figure 2 shows the individual *t*11–8:1/*t*11–18:1 ratio in the intramuscular fat of lambs that received both diets, with *t*10–18:1/*t*11–18:1 ≥ 1 considered as an indicator of the *t*10-shift occurrence [2]. As expected, in all lambs fed the conventional diet showed a higher proportion of *t*10–18:1 than *t*11–18:1 in intramuscular fat, suggesting the predominance of rumen BH pathway that favouring *t*10–18:1 production instead *t*11–18:1. Although it is not completely clear which factors promoted the *t*10–18:1 BH pathway, its occurrence has been associated with a higher diet starch content and a lower ruminal pH [4], as observed in our control diet. Conversely, the experimental diet drastically increased the intramuscular fat proportions of *t*11–18:1 (+688%) while the proportions of *t*10–18:1 were not affected by the diet. Such results suggest that the experimental diet did not inhibit the *t*10–18:1 production in the rumen but promoted the *t*11–18:1 rumen BH pathway. Similar outcomes have been reported by Costa et al. [55] and Gómez-Cortés et al. [56] in lambs fed high-fibre and low-starch diets.

As mentioned above, the induction of the *t*10-shift has been associated with higher diet starch content and lower ruminal pH [4]. However, the isolated effect of each of these factors has been difficult to establish. The occurrence of the *t*10-shift at low rumen pH in lambs fed low-starch diets was reported by Costa et al. [55] and Santos-Silva et al. [9], suggests that ruminal pH can interfere with *t*-10 18:1 production regardless of the dietary starch content. Incorporating 2% sodium bicarbonate as an alkalinizing agent in a high-forage diet limits the occurrence of *t*10-shift and completely prevents it when chopped forage is used instead of ground forage [40]. To avoid the *t*10-shift, in addition to reducing starch levels in the diet, we also incorporated 2% sodium bicarbonate in the experimental diet. Nevertheless, the experimental diet was not able to completely prevent the occurrence of *t*10-shift, once a reduced number of animals presented *t*10–18:1/*t*11–18:1 ≥ 1 in intramuscular fat (Figure 2). The variability in the occurrence of *t*10-shift among animals fed similar diets, even in high-fibre and low-starch diets, is consistent with other reports [9,40]. However, in high-fibre and low-starch diets, the variability of *t*10–18:1/*t*11–18:1 ratio is lower than in high-grain diets, as we also verified in the present work (Figure 2). The factors underlying individual variability in susceptibility to the *t*10-shift are still poorly understood, but are likely related to both rumen microbial ecology and host-specific animal factors [2].

As expected, higher intramuscular concentrations of *t*11–18:1 and *c*9,*t*11–18:2 were observed in lambs fed the experimental diet, as the predominance of *t*11–18:1 rumen BH is characteristic of pasture and high-forage diets [2]. Cellulolytic bacteria are particularly involved in the ruminal synthesis of *t*11–18:1 and *c*9,*t*11–18:2 [57], and the higher fibre content in the experimental diet would have promoted this ruminal microbial community. Supporting this, the changes observed in the intramuscular fat composition of odd- and branched-chain fatty acids (OBCFA) also point toward shifts in the ruminal ecosystem. As odd- and branched-chain fatty acids (OBCFA) are primarily produced from ruminal microbial synthesis, their profile in tissues and milk is used as a biomarker of rumen function and microbial ecosystem [58,59]. Accordingly, the increase in iso-15:0 and the decrease of 15:0, 17:0, and anteiso-17:0 in the experimental diet suggest an increase in cellulolytic bacteria and a decrease in amylolytic bacteria [59]. Moreover, the 15:0 and 17:0 reduction with the experimental diet is consistent with the low starch content in the diet, as these FAs are synthesized from propionate by rumen bacteria [59]. Oliveira et al. [38] also reported a 15:0 and 17:0 decrease in lamb intramuscular fat by reducing the starch content in diets. On the other hand, starch-rich diets depressed the iso-15:0 in milk fat [58].

Dietary lipid supplementation with sources rich in PUFA, as soybean oil, has been used to provide more substrate for rumen BH, and, thus, increase the availability of BHI for tissue deposition, as we observed in the experimental diet, where the proportions of both BHI 18:1 and BHI 18:2 increase in intramuscular fat. When the basal diet promotes the *t*11–18:1 pathway, as seems to occur with the experimental diet, the PUFA overload in the rumen allows a high ruminal production of healthy BHI, such as *t*11–18:1 and *c*9,*t*11–18:2. This is related to the action of the stearol-CoA desaturase (SCD) enzyme [60]. The levels of *c*9,*t*11–18:2 in lamb intramuscular fat are optimized when the following 3 aspects are maximized: i) rumen supply of *t*11–18:1 and *c*9,*t*11–18:2, ii) the endogenous conversion of *t*11–18:1 into *c*9,*t*11–18:1, and iii) fat deposition [2]. In the present study, the experimental diet significantly increased the *c*9,*t*11–18:2 levels (+398%) in the intramuscular fat of lambs. High-starch diets, such as those used in the intensive finishing phase, have been linked with greater deposition of intramuscular fat, as well as to upregulation of SCD [61]. Both intramuscular fat deposition and SCD activity, assessed by calculation of SCDi17, were not affected by diet. On the other hand, the experimental diet induced a higher level of *t*11–18:1 in intramuscular fat, suggesting that the increased *c*9,*t*11–18:2 level induced by this diet is due to increased ruminal production of *c*9,*t*11–18:2 and *t*11–18:1, and consequently higher availability of *c*9,*t*11–18:2 to direct deposition in tissues and higher availability of precursor (*t*11–18:1) to endogenous conversion.

Supplementing diets with PUFA also increases the levels of dietary PUFA that pass through the rumen unchanged. Supplementation of the experimental diet with soybean oil increased the PUFA proportions in intramuscular fat, mainly due to an increase of 18:2n-6 (+84%) and 18:3n-3 (+77%), consistent with their increased dietary intake. However, both n-6 LC-PUFA and n-3 LC-PUFA were not positively related to higher availability of precursors, and levels of 20:5n-3 and 22:6n-3 even decreased in the experimental diet. The n-6 and n-3 LC-PUFA can be synthesized endogenously from 18:2n-6 and 18:3n-3, respectively, through various desaturation and elongation reactions [62]. The enzymes involved in these biosynthesis pathways competitively convert the n-3 and n-6 PUFA, and the conversion of 18:3n-3 into LC-PUFA is negatively impacted by 18:2n-6 levels in the diet [62], which helps explain the reduction in n-3 LC-PUFA in intramuscular fat in the experimental diet. Both 20:5n-3 and 22:6n-3 are widely recognized for health-promoting effects, and adequate dietary intake is considered particularly important for the prevention and treatment of various diseases [63]. To ensure the supply of human nutritional requirements in 20:5n-3 and 22:6n-3 (250 mg per day) [64], the enrichment of foods with these n-3 LC-PUFA has been advocated as desirable. Therefore, the reduction in 20:5n-3 and 22:6n-3 levels in intramuscular fat induced by the experimental diet is an undesirable outcome from a nutritional standpoint. Moreover, the imbalance between n-6 PUFA and n-3 PUFA in food is not favourable for human health, since excessive consumption of n-6 PUFA or a high n-6 PUFA/n-3 PUFA ratio is strongly linked to the development of chronic diseases, such as cancer, cardiovascular, inflammatory, and autoimmune diseases [65]. The control diet already resulted in an n-6 PUFA/n-3 PUFA ratio greater than the recommended value of 4–5/1 on the human diet [65], and high levels of 18:2n-6 in the experimental diet still created a greater imbalance in the n-6 PUFA/n-3 PUFA ratio (Table 8). The use of other lipid supplementation strategies may be a good approach to enhance the n-3 LC-PUFA content and reduce the n-6 PUFA/n-3 PUFA ratio in lamb meat. For instance, dietary supplementation with a blend of vegetable oils rich in 18:2n-6 and 18:3n-3 (e.g., blend of sunflower and linseed oils) is a good strategy to obtain lamb meat enriched in both *c*9,*t*11–18:2 and n-3 LC-PUFA, and reduce the n-6 PUFA/n-3 PUFA ratio to values below 4 [66]. Utilization of n-3 LC-PUFA sources such as fish oil or algae is another approach to increasing n-3 LC-PUFA levels in lamb meat [67,68].

Although the experimental diet did not increase n-3 LC-PUFA and even resulted in an imbalanced n-6 PUFA/n-3 PUFA ratio in the meat, lambs fed this diet produced meat containing 7.8 and 4.8-fold more *t*11–18:1 and *c*9,*t*11–18:2, respectively, compared with meat from lambs fed cereal-rich concentrate diets, when considering the amounts supplied by fresh meat. These FA are recognized for their potential health-promoting properties, including anti-inflammatory, anticarcinogenic, antiatherosclerotic, and antidiabetic effects [6,8], making their enrichment in lamb meat particularly desirable. Furthermore, the experimental diet improved the health-promoting index and reduced the atherogenicity index, indicating a FA composition more favourable to human health compared to meat obtained through a conventional feeding system.

## 5. Conclusions

The experimental diet, composed of 40% of high-quality forage, with partial replacement of cereals by low-starch agro-industrial by-products and supplemented with vegetable oil rich in PUFA, can be used in lamb feedlots, enhancing the intramuscular fat content of healthy FA (*t*11–18:1, *c*9,*t*11–18:2 and 18:3n-3) and improving the health-promoting index without negatively affecting the animals’ ADG or the overall meat quality. However, this feeding strategy did not decrease the levels of *t*10–18:1 and increased the n-6 PUFA/n-3 PUFA ratio in intramuscular fat, in addition to raising feeding costs. Thus, the experimental diet should be improved to allow simultaneous lamb meat enhancement in *c*9,*t*11–18:2 and n-3 PUFA, reducing the n-6 PUFA/n-3 PUFA ratio, without compromising profitability.

## Figures and Tables

**Table 1:** Ingredients of the high-fibre, low-starch, and lipid-supplemented diet (experimental diet).

Ingredients	g/kg
Maize	86
Wheat	90
Dehydrated citrus pulp	55
Dehydrated sugar beet pulp	60
Soybean hulls	60
Soybean meal	30
Sunflower meal	110
Soybean oil	60
Dehydrated lucerne	400
Calcium Carbonate	13
Sodium bicarbonate	9
Dicalcium phosphate	20
Salt	4
Premix ^1^	3

^1^ Premix composition/kg—vitamins A: 4,000,000 UI, D3: 1,100,000 UI, E: 7500 mg, B1 and B2: 250 mg; trace elements zinc: 35 g, Iron: 12.5 g, copper: 250 mg, manganese: 17.5 g, iodine: 200 mg, cobalt: 250 mg, selenium: 100 mg.

**Table 2:** Chemical composition, antioxidant activity, and fatty acid profile of the concentrate-based diet (control) or a high-fibre, low-starch, and high-lipid diet (experimental).

	Diets	
	Control	Experimental
*Chemical composition (g/kg dry matter)*		
Dry matter ^1^ (DM)	896	919
Crude protein	177	150
Ether extract	18.9	77.0
Crude fibre	34.5	168
NDF ^2^	180	343
ADF ^3^	61.5	224
ADL ^4^	9.79	44.9
Sugar	77.4	64.2
Starch	509	173
Ash	69.2	105
Total fatty acids	23.1	60.7
Metabolizable energy (kcal/kg DM) ^5^	2838	2224
Total phenols (g TAE/kg DM) ^6^	2.22	5.68
*Antioxidant activity*		
FRAP ^7^	13.1	70.9
TEAC ^8^	11.4	65.1
*Fatty acid profile (g/100 g total fatty acids)*		
14:0	0.61	0.18
16:0	34.9	15.0
18:0	3.99	4.48
*c*9–18:1	33.9	23.3
*c*11–18:1	1.02	1.51
18:2n-6	23.3	48.0
18:3n-3	1.17	6.44

^1^ Dry matter expressed as g/kg, calculated as the mass of the feed after the moisture has been removed through oven drying; ^2^ Neutral detergent fibre; ^3^ Acid detergent fibre; ^4^ Acid detergent Lignin; ^5^ Metabolizable energy calculated as described by Sauvant et al. [11]; ^6^ Total phenols content expressed as tannic acid equivalents (TAE); ^7^ Ferric reducing antioxidant power, μmol of Fe2+ equivalents/g dry matter; ^8^ Trolox equivalent antioxidant capacity, μmol of trolox equivalents/g dry matter.

**Table 3:** Effects of the diet on indicators of productive performance and nutrient intake from lambs fed a concentrate-based diet (control) or a high-fibre, low-starch and high-lipid diet (experimental).

	Diets		SEM ^1^	*p* Values
	Control	Experimental		
*Growth performance*				
Initial live weight (kg) ^2^	21.5	21.0	0.398	-
Slaughter live weight (kg) ^2,3^	31.8	31.1	0.41	0.228
Average daily gain (g/d) ^2^	341	325	12.2	0.384
Feed conversion ratio ^4,5^	3.61	4.72	0.108	0.002
Feed cost/kg of weight gain (€) ^4^	1.73	2.09	0.048	0.006
*Daily intake* (g/day) ^4^				
Dry matter	1089	1354	18.5	<0.001
Crude protein	193	203	3.0	0.031
Ether extract	20.6	104	1.05	<0.001
Crude fibre	37.6	228	2.29	<0.001
NFD ^6^	196	465	5.14	<0.001
ADF ^7^	67.0	304	3.10	<0.001
ADL ^8^	10.7	60.8	0.61	<0.001
Sugar	84.3	86.9	1.32	0.184
Starch	555	235	7.0	<0.001
Ash	75.4	143	1.66	<0.001
Metabolizable energy (kcal/day)	3091	3010	47.1	0.240
Total phenols	2.40	7.69	0.081	<0.001
*Fatty acids*				
14:0	0.15	0.15	0.002	0.456
16:0	8.77	12.3	0.160	<0.001
18:0	1.01	3.68	0.038	<0.001
*c*9–18:1	8.52	19.2	0.214	<0.001
*c*11–18:1	0.26	1.24	0.013	<0.001
18:2n-6	5.84	39.4	0.395	<0.001
18:3n-3	0.37	5.28	0.052	<0.001
				
Rumen pH	5.60	6.93	0.097	<0.001

^1^ Standard error of the mean; ^2^ Data from 45 lambs per diet (15 lambs per pen and 3 pens per diet); ^3^ Adjusted for initial live weight; ^4^ Data from 3 pens per diet, each with 15 lambs; ^5^ Feed conversion ratio = kg dry matter intake/kg weight increase; ^6^ Neutral detergent fibre; ^7^ Acid detergent fibre; ^8^ Acid detergent Lignin.

**Table 4:** Effects of the diet on carcass quality, shoulder composition, and meat physicochemical and sensorial characteristics from lambs fed a concentrate-based diet (control) or a high-fibre, low-starch and high-lipid diet (experimental).

	Diets		SEM ^1^	*p* Values
	Control	Experimental		
*Carcass traits* ^2^				
Hot carcass weight (kg) ^3^	15.4	15.6	0.24	0.586
Cold carcass weight (kg) ^3^	14.9	15.1	0.25	0.589
Dressing (%) ^4^	48.2	48.8	0.71	0.252
Kidney knob channel fat (%) ^5^	1.82	2.50	0.104	<0.001
*Shoulder composition (g/kg)* ^2^				
Muscle ^5^	600	576	12.4	0.240
Bone ^5^	221	223	10.5	0.912
Subcutaneous fat ^5^	72	84	10.6	0.476
Intermuscular fat ^5^	104	111	11.5	0.697
*Meat chemical and physical characteristics* ^2^				
Dry matter (g/kg)	245	243	4.5	0.713
Crude protein (g/kg)	207	206	3.0	0.846
Intramuscular fat (g/kg)	12.3	12.3	0.93	0.977
pH	5.66	5.49	0.035	0.032
Shear force (N) ^6^	33.6	40.2	4.59	0.355
Cooking loss (g/100 g) ^6^	29.8	31.2	1.18	0.470
*Meat sensory attributes* ^2^				
Juiciness	4.83	4.81	0.199	0.830
Tenderness	5.34	5.04	0.161	0.121
Odour intensity	2.29	2.40	0.303	0.430
Flavour intensity	2.25	2.32	0.404	0.704
Flavour acceptability	5.44	5.37	0.109	0.380
Overall acceptability	5.34	5.23	0.098	0.308

^1^ Standard error of the mean; ^2^ Data from 12 lambs per diet (4 lambs per pen and 3 pens per diet); ^3^ Adjusted for initial live weight; ^4^ Dressing = (hot carcass weight/slaughter live weight) × 100; ^5^ Adjusted for hot carcass weight; ^6^ Adjusted for meat pH.

**Table 5:** Effects of the diet and storage time on colour and lipid stability of meat from lambs fed a concentrate-based diet (control) or a high-fibre, low-starch and high-lipid diet (experimental).

	Diet		SEM ^1^	Time (Days)			SEM ^1^	*p* Values	
	Control	Experimental		0	4	7		Diet	Time
*Colour parameters* ^2,3^									
L*	45.8	45.6	0.59	44.9	46.2	46.0	0.56	0.801	0.113
a*	14.8	13.7	0.37	15.0 ^b^	14.6 ^b^	13.1 ^a^	0.38	0.084	<0.001
b*	10.0	9.66	0.162	7.19 ^a^	11.2 ^b^	11.1 ^b^	0.198	0.120	<0.001
C*	18.0	16.9	0.28	16.7 ^a^	18.4 ^b^	17.3 ^b^	0.32	0.052	<0.001
H*	33.8	35.4	0.76	25.5 ^a^	37.6 ^b^	40.7 ^c^	0.87	0.230	<0.001
∆E	4.96	4.94	0.347	-	4.72	5.18	0.308	0.981	0.215
*Lipid oxidation* ^2^									
TBARS ^4^	0.60	0.68	0.094	0.03 ^a^	0.64 ^b^	1.25 ^c^	0.085	0.606	<0.001

^1^ Standard error of the mean; ^2^ Data from 12 lambs per diet (4 lambs per pen and 3 pens per diet); ^3^ L*—lightness; a*—redness; b*- yellowness; C*—chroma; H*—hue angle; ∆E—colour stability index; ^4^ Thiobarbituric acid reactive substances, expressed as mg of malonaldehyde/kg of meat; Values with different superscripts are significantly different (*p* < 0.05).

**Table 6:** Effects of the diet on fatty acid (FA) profile in intramuscular fat of lambs fed a concentrate-based diet (control) or a high-fibre, low-starch and high-lipid diet (experimental).

	Diets		SEM ^1^	*p* Values
	Control	Experimental		
*Fatty acid profile (mg/g total fatty acids)* ^2^				
LC-SFA ^3^				
10:0	0.88	0.81	0.064	0.525
12:0	1.24	1.08	0.137	0.453
14:0	22.5	19.8	1.23	0.194
15:0	4.09	2.63	0.204	0.007
16:0	226	211	4.1	0.058
17:0	15.5	7.21	0.702	0.001
18:0	166	165	4.9	0.891
20:0	1.13	1.16	0.052	0.750
22:0	0.58	0.64	0.061	0.532
BCFA ^4^				
iso-14:0	0.22	0.26	0.046	0.568
iso-15:0	0.40	0.56	0.023	0.006
iso-16:0	0.68	0.75	0.036	0.255
iso-17:0	1.80	1.44	0.106	0.073
iso*-*18:0	0.71	0.46	0.031	<0.001
anteiso-15:0	0.65	0.80	0.040	0.064
anteiso-17:0	3.17	2.24	0.202	0.031
*cis*-MUFA ^5^				
*c*9–14:1	0.51	0.31	0.057	0.065
*c*7–16:1	2.75	2.36	0.085	0.030
*c*9–16:1	13.6	6.46	0.766	0.003
*c*9–17:1	9.13	3.16	0.354	<0.001
*c*9–18:1	343	243	8.0	<0.001
*c*11–18:1	16.4	12.2	0.93	0.033
*c*19–19:1	0.62	0.47	0.036	0.024
n-6 PUFA ^6^				
18:2n-6	60.9	112	7.23	0.007
18:3n-6	0.67	0.40	0.058	0.032
20:2n-6	0.50	0.93	0.086	0.023
20:3n-6	2.19	1.69	0.212	0.175
20:4n-6	21.5	18.3	2.37	0.403
22:4n-6	1.99	1.79	0.356	0.713
22:5n-6	0.49	0.61	0.092	0.324
n-3 PUFA ^7^				
18:3n-3	3.92	6.95	0.205	<0.001
20:5n-3	2.38	1.33	0.171	0.012
22:5n-3	4.38	3.48	0.389	0.179
22:6n-3	1.06	0.53	0.049	0.002
20:3n-9	3.22	1.79	0.195	0.007
Dimethyl acetals (DMA)				
DMA 16:0	1.19	1.22	0.291	0.931
DMA 18:0	0.67	0.85	0.156	0.440
DMA 18:1	1.17	1.17	0.142	0.994
Partial sums and ratios				
LC-SFA^3^	438	409	6.5	0.034
iso-BCFA^4^	3.72	3.45	0.201	0.361
anteiso-BCFA ^4^	3.82	3.04	0.241	0.082
BCFA ^4^	7.54	6.49	0.403	0.081
*cis*-MUFA ^4^	393	308	7.2	<0.001
*trans*-MUFA ^5^	41.7	92.1	1.08	<0.001
MUFA ^5^	435	400	6.8	0.023
n-6 PUFA ^6^	88.0	136	10.37	0.031
n-6 LC-PUFA ^8^	26.5	23.0	3.12	0.473
n-3 PUFA ^7^	11.7	12.3	0.69	0.599
n-3 LC -PUFA ^9^	7.82	5.35	0.554	0.034
PUFA ^10^	108	171	11.1	0.016
SCDi-17 ^11^	29.7	30.2	3.23	0.950

^1^ Standard error of the mean; ^2^ Data from 12 lambs per diet (4 lambs per pen and 3 pens per diet); ^3^ Linear chain saturated fatty acids; ^4^ Branched chain fatty acids; ^5^ Monounsaturated fatty acids; ^6^ Polyunsaturated fatty acids n-6; ^7^ Polyunsaturated fatty acids n-3; ^8^ Long chain polyunsaturated fatty acids n-6; ^9^ Long chain polyunsaturated fatty acids n-3; ^10^ Polyunsaturated fatty acids; ^11^ SCDi-17 = (*c*9–17:1/(*c*9–17:1 + 17:0) × 100.

**Table 7:** Effects of the diet on biohydrogenation intermediate (BHI) in intramuscular fat of lambs fed a concentrate-based diet (control) or a high-fibre, low-starch and high-lipid diet (experimental).

	Diets		SEM ^1^	*p* Values
	Control	Experimental		
*Fatty acid profile of biohydrogenation intermediate (mg/g total fatty acids)* ^2^				
18:1 isomers				
*t*4–18:1	0.07	0.44	0.026	<0.001
*t*5–18:1	0.22	0.67	0.026	<0.001
*t*6-/*t*7-/*t*8–18:1	2.97	6.58	0.145	<0.001
*t*9–18:1	3.05	5.27	0.101	<0.001
*t*10–18:1	26.6	28.4	4.31	0.783
*t*11–18:1	4.01	31.6	3.95	0.008
*t*12–18:1	3.66	11.0	0.390	<0.001
*t*15–18:1	1.30	4.02	0.670	0.034
*t*16–18:1 ^3^	0.69	2.97	0.216	0.002
*c*12–18:1	4.89	33.1	2.516	0.001
*c*13–18:1	1.12	0.85	0.110	0.166
*c*15–18:1	0.44	1.11	0.042	<0.001
*c*16–18:1	0.66	4.17	0.270	<0.001
18:2 isomers				
*c*9,*t*13-/*c*9,*t*14–18:2 ^4^	1.13	3.56	0.480	0.023
*t*8,*c*13-/*c*9,*t*15–18:2	0.54	1.82	0.459	0.124
*c*9,*t*12–18:2	0.65	1.16	0.079	0.011
*t*9,*c*12–18:2	0.90	2.17	0.144	0.003
*t*11,*c*15–18:2 ^5^	0.71	2.90	0.231	0.003
*c*9,*t*11–18:2 ^6^	1.94	9.66	1.076	0.007
BHI 18:1	41.5	90.9	1.03	<0.001
BHI 18:2	5.43	21.3	1.275	<0.001
*t*10–18:1/*t*11–18:1ratio	7.98	1.17	1.078	0.011

^1^ Standard error of the mean; ^2^ Data from 12 lambs per diet (4 lambs per pen and 3 pens per diet); ^3^ Coelutes with c14–18:1 as minor isomer; ^4^ Coelutes with cyclo-17; ^5^ Coelutes with *t*10,*c*15–18:2; ^6^ Coelutes with *t*7,*c*9- and *t*8,*c*10–18:2 as minor isomers.

**Table 8:** Effects of the diet on fatty acid (FA) composition in intramuscular fat of lambs fed a concentrate-based diet (control) or a high-fibre, low-starch and high-lipid diet (experimental).

	Diets		SEM ^1^	*p* Value
	Control	Experimental		
*Fatty acid composition (mg/100 g muscle)* ^2^				
14:0	23.7	22.4	2.23	0.691
16:0	232	237	19.7	0.857
18:0	174	185	14.8	0.635
*c*9–16:1	13.7	7.21	1.308	0.025
*c*9–18:1	356	274	28.6	0.112
*t*10–18:1	24.8	34.0	6.23	0.357
*t*11–18:1	4.50	34.9	4.39	0.008
*c*9,*t*11–18:2	2.22	10.7	1.19	0.007
18:2n-6	56.2	120	2.24	<0.001
20:4n-6	19.7	18.2	0.84	0.259
18:3n-3	4.07	7.74	0.276	<0.001
20:5n-3	2.43	1.52	0.160	0.016
22:5n-3	4.26	3.79	0.158	0.107
22:6n-3	1.08	0.58	0.038	<0.001
Partial sums and indices				
LC-SFA ^3^	453	459	37.0	0.922
BCFA ^4^	7.86	7.23	0.778	0.601
*cis*-MUFA ^5^	406	345	31.9	0.248
*trans*-MUFA ^5^	40.3	104	4.71	<0.001
MUFA ^5^	446	449	36.2	0.955
n-6 PUFA ^6^	81.1	143	3.25	<0.001
n-6 LC-PUFA ^7^	24.9	23.3	1.27	0.429
n-3 PUFA ^8^	11.8	13.6	0.54	0.077
n-3 LC-PUFA ^9^	7.76	5.89	0.309	0.013
n-6 PUFA/n-3 PUFA ^6,8^	7.62	11.2	0.667	0.020
PUFA ^10^	102	183	3.9	<0.001
HH ^11^	1.91	2.02	0.061	0.264
Atherogenicity index ^12^	0.58	0.51	0.017	0.037
Thrombogenicity index ^12^	1.40	1.30	0.038	0.127
Health-promoting index ^13^	1.72	1.98	0.056	0.035

^1^ Standard error of the mean; ^2^ Data from 12 lambs per diet (4 lambs per pen and 3 pens per diet); ^3^ Linear chain saturated fatty acids; ^4^ Branched chain fatty acids; ^5^ Monounsaturated fatty acids; ^6^ Polyunsaturated fatty acids n-6; ^7^ Long chain polyunsaturated fatty acids n-6; ^8^ Polyunsaturated fatty acids n-3; ^9^ Long chain polyunsaturated fatty acids n-3; ^10^ Polyunsaturated fatty acids; ^11^ hypocholesterolemic/hypercholesterolemic ratio calculated as described by Santos-Silva et al. [29]; ^12^ atherogenicity and thrombogenicity indices calculated according to Ulbricht and Southgate [30]; ^13^ health-promoting index is calculated according to Chen et al. [31].

## Data Availability

The original contributions presented in this study are included in the article. Further inquiries can be directed to the corresponding author.

## Abbreviations

The following abbreviations are used in this manuscript:

ADF	Acid detergent fibre
ADG	Average daily gain
ADL	Acid detergent lignin
BCFA	Branched-chain fatty acids
BH	Ruminal biohydrogenation
BHI	Ruminal biohydrogenation intermediates
DM	Dry matter
DMA	Dimethyl acetals
FA	Fatty acid
FRAP	Ferric reducing antioxidant power
LC-SFA	Linear chain saturated fatty acids
LL	*Longissimus lomborum*
LT	*Longissimus thoracis*
MDA	Malondialdehyde
MUFA	Monounsaturated fatty acids
NDF	Neutral detergent fibre
OBCFA	Odd- and branched-chain fatty acids
PUFA	Polyunsaturated fatty acids
SFA	Saturated fatty acids
TAE	Tannic acid equivalents
TEAC	Trolox equivalent antioxidant capacity
TBARS	Thiobarbituric acid reactive substances

## References

[1] Campo M.M., Mur L., Fugita C.A., Sañudo C. (2016). Current strategies in lamb production in Mediterranean areas. Anim. Front..

[2] Bessa R.J.B., Alves S.P., Santos-Silva J. (2015). Constraints and potentials for the nutritional modulation of the fatty acid composition of ruminant meat. Eur. J. Lipid Sci. Technol..

[3] Aldai N., de Renobales M., Barron L.J.R., Kramer J.K.G. (2013). What are the trans fatty acids issues in foods after discontinuation of industrially produced trans fats? Ruminant products, vegetable oils, and synthetic supplements. Eur. J. Lipid Sci. Technol..

[4] Alves S.P., Vahmani P., Mapiye C., McAllister T.A., Bessa R.J.B., Dugan M.E.R. (2021). *Trans*-10 18:1 in ruminant meats: A review. Lipids.

[5] Dugan M.E.R., Salazar V., Rolland D.C., Vahmani P., Aalhus J.L., López-Campos Ó., Prieto N., Juárez M. (2019). Retail lamb fat composition in western Canada. Can. J. Anim. Sci..

[6] Vahmani P., Xu Y., Dugan M.E.R., Hackmann T.J. (2024). *Trans*-10 shifted ruminal biohydrogenation and its implications for ruminant milk and meat fat content and quality. Can. J. Anim. Sci..

[7] Chikwanha O.C., Vahmani P., Muchenje V., Dugan M.E.R., Mapiye C. (2018). Nutritional enhancement of sheep meat fatty acid profile for human health and wellbeing. Food Res. Int..

[8] Vahmani P., Ponnampalam E.N., Kraft J., Mapiye C., Bermingham E.N., Watkins P.J., Proctor S.D., Dugan M.E.R. (2020). Bioactivity and health effects of ruminant meat lipids. Invited Review. Meat Sci..

[9] Santos-Silva J., Francisco A., Alves S.P., Portugal P., Dentinho T., Almeida J., Soldado D., Jerónimo E., Bessa R.J.B. (2019). Effect of dietary neutral detergent fibre source on lambs growth, meat quality and biohydrogenation intermediates. Meat Sci..

[10] European Union (2010). Diretive 2010/63/EU of the European Parliament and of the Council of 22 September 2010 on the protection of animals used for scientific purposes. Off. J. Eur. Union.

[11] Sauvant D., Perez J.M., Tran G. (2002). Tables de Composition et de Valeur Nutritive des Matières Premières Destinées aux Animaux d’Élevage: Porcs, Volailles, Bovins, Ovins, Caprins, Lapins, Chevaux, Poisons.

[12] European Commission (2011). Community Scale for the Classification of Carcases of Ovine Animals.

[13] (1999). Animal Feeding Stuffs—Determination of Moisture and the Other Volatile Matter Content.

[14] (2002). Animal Feeding Stuffs—Determination of Crude Ash.

[15] (1999). Animal Feeding Stuffs—Determination of Fat Content.

[16] (1997). Animal Feeding Stuffs—Determination of Nitrogen Content and Calculation of Crude Protein Content—Kjeldhal Method.

[17] Clegg K.M. (1956). The application of the anthrone reagent to the estimation of starch in cereals. J. Sci. Food Agric..

[18] Goering H.K., Van Soest P.J. (1970). Forage fiber analyses (apparatus, reagents, procedures, and some applications). USDA-ARS Agricultural Handbook.

[19] Makkar H.P.S. (2003). Quantification of Tannins in Tree and Shrub Foliage. A Laboratory Manual.

[20] Luciano G., Vasta V., Monahan F.J., López-Andrés P., Biondi L., Lanza M., Priolo A. (2011). Antioxidant status, colour stability and myoglobin resistance to oxidation of longissimus dorsi muscle from lambs fed a tannin-containing diet. Food Chem..

[21] Sukhija P.S., Palmquist D.L. (1988). Rapid method for determination of total fatty acid content and composition of feedstuffs and feces. J. Agric. Food Chem..

[22] Vítor A.C.M., Francisco A.E., Silva J., Pinho M., Huws S.A., Santos-Silva J., Bessa R.J.B., Alves S.P. (2021). Freeze-dried Nannochloropsis oceanica biomass protects eicosapentaenoic acid (EPA) from metabolization in the rumen of lambs. Sci. Rep..

[23] (1997). Meat and Meat Products—Determination of Moisture Content.

[24] (1999). Meat and Meat Products. Measurement of pH—Reference Method.

[25] Folch J., Lees M., Stanley G.H.S. (1957). A simple method for the isolation and purification of total lipides from animal tissues. J. Biol. Chem..

[26] Cruz-Hernandez C., Deng Z., Zhou J., Hill A.R., Yurawecz M.P., Delmonte P., Mossoba M.M., Dugan M.E.R., Kramer J.K.G. (2004). Methods for analysis of conjugated linoleic acids and trans-18:1 isomers in dairy fats by using a combination of gas chromatography, silver-ion thin-layer chromatography/gas ghromatography, and silver-ion liquid chromatography. J. AOAC Int..

[27] (1993). Sensory Analysis—General Guidance for the Selection, Training and Monitoring of Assessors—Part 1: Selected Assessors.

[28] Grau A., Guardiola F., Boatella J., Barroeta A., Codony R. (2000). Measurement of 2-thiobarbituric acid values in dark chicken meat through derivative spectrophotometry:  Influence of various parameters. J. Agric. Food Chem..

[29] Santos-Silva J., Mendes I.A., Portugal P.V., Bessa R.J.B. (2004). Effect of particle size and soybean oil supplementation on growth performance, carcass and meat quality and fatty acid composition of intramuscular lipids of lambs. Livest. Prod. Sci..

[30] Ulbricht T.L.V., Southgate D.A.T. (1991). Coronary heart disease: Seven dietary factors. Lancet.

[31] Chen S., Bobe G., Zimmerman S., Hammond E.G., Luhman C.M., Boylston T.D., Freeman A.E., Beitz D.C. (2004). Physical and sensory properties of dairy products from cows with various milk fatty acid compositions. J. Agric. Food Chem..

[32] Zhang Z., Li F., Li F., Wang Z., Guo L., Weng X., Sun X., He Z., Meng X., Liang Z. (2025). Influence of dietary forage neutral detergent fiber on ruminal fermentation, chewing activity, nutrient digestion, and ruminal microbiota of Hu sheep. Animals.

[33] Hess B.W., Moss G.E., Rule D.C. (2008). A decade of developments in the area of fat supplementation research with beef cattle and sheep. J. Anim. Sci..

[34] Jenkins T.C. (1993). Lipid Metabolism in the Rumen. J. Dairy Sci..

[35] Manso T., Bodas R., Castro T., Jimeno V., Mantecon A.R. (2009). Animal performance and fatty acid composition of lambs fed with different vegetable oils. Meat Sci..

[36] Jerónimo E., Alves S.P., Martins S.V., Prates J.A.M., Bessa R.J.B., Santos-Silva J. (2010). Effect of sodium bentonite and vegetable oil blend supplementation on growth, carcass quality and intramuscular fatty acid composition of lambs. Anim. Feed Sci. Technol..

[37] Boles J.A., Kott R.W., Hatfield P.G., Bergman J.W., Flynn C.R. (2005). Supplemental safflower oil affects the fatty acid profile, including conjugated linoleic acid, of lamb. J. Anim. Sci..

[38] Oliveira M.A., Alves S.P., Santos-Silva J., Bessa R.J.B. (2017). Effect of dietary starch level and its rumen degradability on lamb meat fatty acid composition. Meat Sci..

[39] Forbes J.M. (2007). Voluntary Food Intake and Diet Selection in Farm Animals.

[40] Francisco A.E., Janíček M., Dentinho T., Portugal A.P.V., Almeida J.M., Alves S.P., Fialho L., Jerónimo E., Bessa R.J.B., Santos-Silva J. (2020). Effects of alfalfa particle size and starch content in diets on feeding behaviour, intake, rumen parameters, animal performance and meat quality of growing lambs. Meat Sci..

[41] Hocquette J.F., Gondret F., Baéza E., Médale F., Jurie C., Pethick D.W. (2010). Intramuscular fat content in meat-producing animals: Development, genetic and nutritional control, and identification of putative markers. Animal.

[42] Watanabe A., Daly C.C., Devine C.E. (1996). The effects of the ultimate pH of meat on tenderness changes during ageing. Meat Sci..

[43] Hopkins D.L., Hegarty R.S., Walker P.J., Pethick D.W. (2006). Relationship between animal age, intramuscular fat, cooking loss, pH, shear force and eating quality of aged meat from sheep. J. Aust. J. Exp. Agric..

[44] Starkey C.P., Geesink G.H., Oddy V.H., Hopkins D.L. (2015). Explaining the variation in lamb longissimus shear force across and within ageing periods using protein degradation, sarcomere length and collagen characteristics. Meat Sci..

[45] Shanks B.C., Wulf D.M., Maddock R.J. (2002). Technical note: The effect of freezing on Warner-Bratzler shear force values of beef longissimus steaks across several postmortem aging periods. J. Anim. Sci..

[46] Luciano G., Monahan F.J., Vasta V., Pennisi P., Bella M., Priolo A. (2009). Lipid and colour stability of meat from lambs fed fresh herbage or concentrate. Meat Sci..

[47] Khliji S., van de Ven R., Lamb T.A., Lanza M., Hopkins D.L. (2010). Relationship between consumer ranking of lamb colour and objective measures of colour. Meat Sci..

[48] Abril M., Campo M.M., Önenç A., Sañudo C., Albertí P., Negueruela A.I. (2001). Beef colour evolution as a function of ultimate pH. Meat Sci..

[49] Domínguez R., Pateiro M., Gagaoua M., Barba F.J., Zhang W., Lorenzo J.M. (2019). A comprehensive review on lipid oxidation in meat and meat products. Antioxidants.

[50] Inserra L., Luciano G., Bella M., Scerra M., Cilione C., Basile P., Lanza M., Priolo A. (2015). Effect of including carob pulp in the diet of fattening pigs on the fatty acid composition and oxidative stability of pork. Meat Sci..

[51] Scerra M., Bognanno M., Foti F., Caparra P., Cilione C., Mangano F., Natalello A., Chies L. (2022). Influence of almond hulls in lamb diets on animal performance and meat quality. Meat Sci..

[52] Natalello A., Priolo A., Valenti B., Codini M., Mattioli S., Pauselli M., Puccio M., Lanza M., Stergiadis S., Luciano G. (2020). Dietary pomegranate by-product improves oxidative stability of lamb meat. Meat Sci..

[53] Ripoll G., Joy M., Muñoz F. (2011). Use of dietary vitamin E and selenium (Se) to increase the shelf life of modified atmosphere packaged light lamb meat. Meat Sci..

[54] Campo M.M., Nute G.R., Hughes S.I., Enser M., Wood J.D., Richardson R.I. (2006). Flavour perception of oxidation in beef. Meat Sci..

[55] Costa M., Alves S.P., Francisco A., Almeida J., Alfaia C.M., Martins S.V., Prates J.A.M., Santos-Silva J., Doran O., Bessa R.J.B. (2017). The reduction of starch in finishing diets supplemented with oil does not prevent the accumulation of *trans*-10 18:1 in lamb meat1. J. Anim. Sci..

[56] Gómez-Cortés P., Galisteo O.O., Ramírez C.A., Blanco F.P., Angel de la Fuente M., Sánchez N.N., Marín A.L.M. (2019). Intramuscular fatty acid profile of feedlot lambs fed concentrates with alternative ingredients. J. Anim. Prod. Sci..

[57] Harfoot C., Hazelwood G. (1997). Lipid metabolism in the rumen. The Rumen Microbial Ecosystem.

[58] Vlaeminck B., Fievez V., Cabrita A.R.J., Fonseca A.J.M., Dewhurst R.J. (2006). Factors affecting odd- and branched-chain fatty acids in milk: A review. Anim. Feed Sci. Technol..

[59] Fievez V., Colman E., Castro-Montoya J.M., Stefanov I., Vlaeminck B. (2012). Milk odd- and branched-chain fatty acids as biomarkers of rumen function—An update. Anim. Feed Sci. Technol..

[60] Griinari J.M., Corl B.A., Lacy S.H., Chouinard P.Y., Nurmela K.V.V., Bauman D.E. (2000). Conjugated linoleic acid is synthesized endogenously in lactating dairy cows by Δ9-desaturase. J. Nutr..

[61] Daniel Z.C.T.R., Wynn R.J., Salter A.M., Buttery P.J. (2004). Differing effects of forage and concentrate diets on the oleic acid and conjugated linoleic acid content of sheep tissues: The role of stearoyl-CoA desaturase. J. Anim. Sci..

[62] Gonzalez-Soto M., Mutch D.M. (2021). Diet regulation of long-chain PUFA synthesis: Role of macronutrients, micronutrients, and polyphenols on Δ-5/Δ-6 desaturases and elongases 2/5. Adv. Nutr..

[63] Troesch B., Eggersdorfer M., Laviano A., Rolland Y., Smith A.D., Warnke I., Weimann A., Calder P.C. (2020). Expert opinion on benefits of long-chain omega-3 fatty acids (DHA and EPA) in aging and clinical nutrition. Nutrients.

[64] FAO (2010). Fats and Fatty Acids in Human Nutrition. FAO—Report of an Expert Consultation.

[65] Mariamenatu A.H., Abdu E.M. (2021). Overconsumption of omega-6 polyunsaturated fatty acids (PUFAs) versus deficiency of omega-3 PUFAs in modern-day diets: The disturbing factor for their “balanced antagonistic metabolic functions” in the Human body. J. Lipids.

[66] Jerónimo E., Alves S.P., Prates J.A.M., Santos-Silva J., Bessa R.J.B. (2009). Effect of dietary replacement of sunflower oil with linseed oil on intramuscular fatty acids of lamb meat. Meat Sci..

[67] Cooper S.L., Sinclair L.A., Wilkinson R.G., Hallett K.G., Enser M., Wood J.D. (2004). Manipulation of the n-3 polyunsaturated fatty acid content of muscle and adipose tissue in lambs. J. Anim. Sci..

[68] Orzuna-Orzuna J.F., Hernández-García P.A., Chay-Canul A.J., Díaz Galván C., Razo Ortíz P.B. (2023). Microalgae as a dietary additive for lambs: A meta-analysis on growth performance, meat quality, and meat fatty acid profile. Small Rumin. Res..

[69] European Union (2016). Directive 2016/679 of the European Parliament and of the Council of 27 April 2016 on the protection of natural persons with regard to the processing of personal data and on the free movement of such data. Off. J. Eur. Union.

[70] Instituto Nacional de Investigação Agrária e Veterinária, Instituto Público (2022). Código de Ética e Conduta 2022–2025.

